# Allyl 2-(2,2-dimethyl-3a,6a-di­hydro­furo[3,2-*d*][1,3]dioxol-5-yl)-4-oxo-4*H*-chromene-3-carboxyl­ate

**DOI:** 10.1107/S1600536813018904

**Published:** 2013-07-13

**Authors:** Zeenat Fatima, Thothadri Srinivasan, Jonnalagadda Naga Siva Rao, Raghavachary Raghunathan, Devadasan Velmurugan

**Affiliations:** a Centre of Advanced Study in Crystallography and Biophysics, University of Madras, Guindy Campus, Chennai 600 025, India; b Department of Organic Chemistry, University of Madras, Guindy Campus, Chennai 600 025, India

## Abstract

In the title compound, C_20_H_18_O_7_, the dioxolane ring adopts an envelope conformation with the dimethyl-substituted C atom as the flap, and its mean plane makes a dihedral angle of 73.25 (2)° with the pyran ring mean plane. The furan ring makes dihedral angles of 67.43 (12) and 6.20 (11)° with the mean plane of the dioxolane and pyran rings, respectively. The O atom attached to the pyran ring deviates by 0.0219 (2) Å from its mean plane. In the crystal, mol­ecules are linked *via* C—H⋯O hydrogen bonds, forming chains along [010] and enclosing *R*_2_^2^(9) loops. They stack along the *a* axis with π–π inter­actions involving the 4*H*-chromene units [centroid–centroid distances of 3.6389 (13) and 3.6555 (13) Å]. The terminal CH_2_=CH- atoms of the allyl acetate group are disordered over two sets of sites with a refined occupancy ratio of 0.717 (6):0.283 (6).

## Related literature

For the biological importance of 4*H*-chromene derivatives, see: Cai (2007 ▶, 2008 ▶); Cai *et al.* (2006 ▶); Caine (1993 ▶); Gabor (1988 ▶); Brooks (1998 ▶); Valenti *et al.* (1993 ▶); Hyana & Saimoto (1987 ▶); Tang *et al.* (2007 ▶).
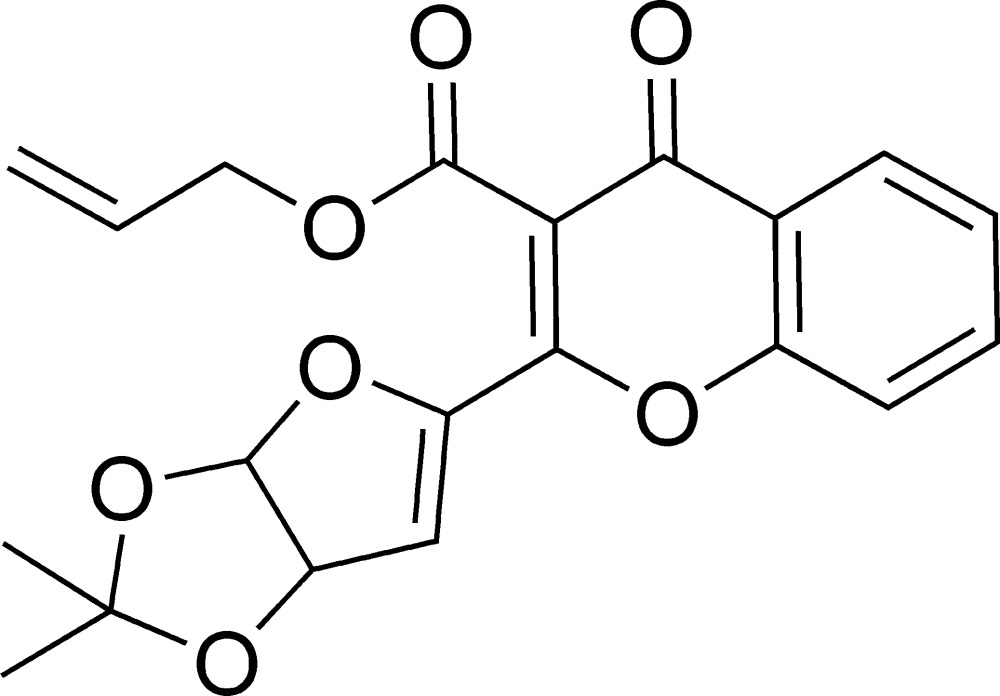


## Experimental

### 

#### Crystal data

C_20_H_18_O_7_
*M* *_r_* = 370.34
Orthorhombic, 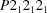

*a* = 6.9461 (6) Å
*b* = 15.5688 (11) Å
*c* = 16.5572 (11) Å
*V* = 1790.5 (2) Å^3^
*Z* = 4
Mo *K*α radiation
μ = 0.11 mm^−1^
*T* = 293 K
0.30 × 0.25 × 0.20 mm

#### Data collection

Bruker SMART APEXII area-detector diffractometer
Absorption correction: multi-scan (*SADABS*; Bruker, 2008 ▶) *T*_min_ = 0.969, *T*_max_ = 0.979
17828 measured reflections
4459 independent reflections
2758 reflections with *I* > 2σ(*I*)
*R*_int_ = 0.072

#### Refinement

*R*[*F* ^2^ > 2σ(*F* ^2^)] = 0.045
*wR*(*F* ^2^) = 0.127
*S* = 1.01
4459 reflections
252 parameters
3 restraints
H-atom parameters constrained
Δρ_max_ = 0.16 e Å^−3^
Δρ_min_ = −0.18 e Å^−3^

### 

Data collection: *APEX2* (Bruker, 2008 ▶); cell refinement: *SAINT* (Bruker, 2008 ▶); data reduction: *SAINT*; program(s) used to solve structure: *SHELXS97* (Sheldrick, 2008 ▶); program(s) used to refine structure: *SHELXL97* (Sheldrick, 2008 ▶); molecular graphics: *ORTEP-3 for Windows* (Farrugia, 2012 ▶); software used to prepare material for publication: *SHELXL97* and *PLATON* (Spek, 2009 ▶).

## Supplementary Material

Crystal structure: contains datablock(s) global, I. DOI: 10.1107/S1600536813018904/su2620sup1.cif

Structure factors: contains datablock(s) I. DOI: 10.1107/S1600536813018904/su2620Isup2.hkl

Supplementary material file. DOI: 10.1107/S1600536813018904/su2620Isup3.cml

Additional supplementary materials:  crystallographic information; 3D view; checkCIF report

## Figures and Tables

**Table 1:** Hydrogen-bond geometry (Å, °)

*D*—H⋯*A*	*D*—H	H⋯*A*	*D*⋯*A*	*D*—H⋯*A*
C11—H11⋯O3^i^	0.93	2.47	3.190 (3)	134
C12—H12⋯O2^i^	0.98	2.54	3.477 (3)	160

Symmetry code: (i) 
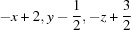
.

**Fig. 1.**
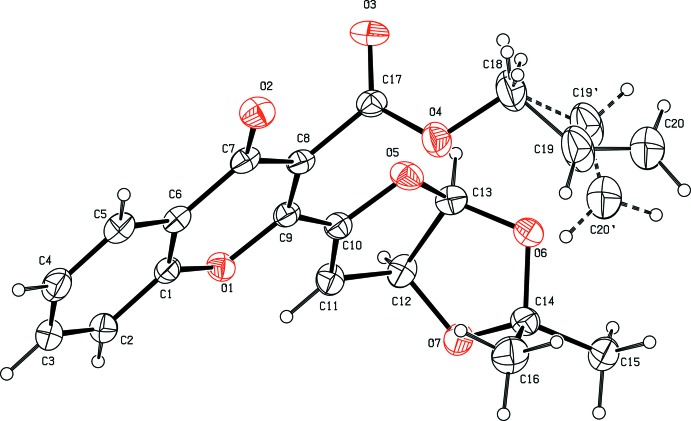
The molecular structure of the title molecule, with atom labelling. The displacement ellipsoids are drawn at the 30% probability level. The position of the disordered terminal atoms (C19' & C20') of the allyl acetate group are shown with dashed bonds.

**Fig. 2.**
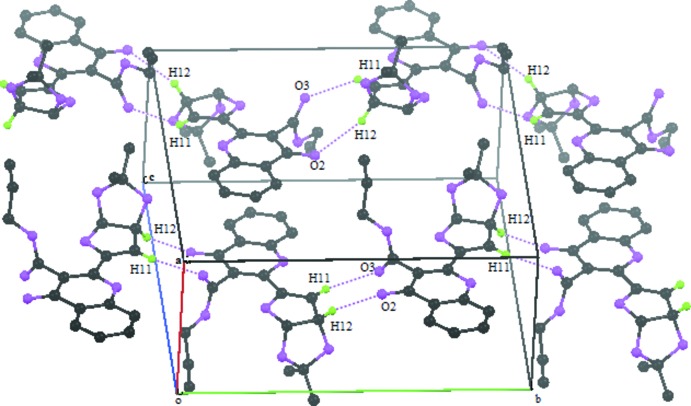
The crystal packing of the title compound, viewed along the a axis. The hydrogen bonds (see Table 1 for details) are shown as dashed lines. H-atoms not involved in hydrogen bonding have been omitted for clarity.

## supplementary crystallographic information

### Comment

4H-Chromenes are biologically important compounds used as synthetic ligands in the design of drugs and discovery processes. They exhibit numerous biological and pharmacological properties, such as anti-viral, anti-fungal, anti-inflammatory, anti-diabetic, cardionthonic, anti-anaphylactic and anti-cancer activity (Cai, 2008, 2007; Cai *et al.*, 2006; Caine, 1993; Gabor, 1988; Brooks, 1998; Valenti *et al.*, 1993; Hyana & Saimoto, 1987; Tang *et al.*, 2007). In view of the different applications of this class of compounds, we have undertaken the synthesis and crystal structure analysis of the title compound.

In the title molecule, Fig. 1, the dioxolane ring adopts an *envelope* conformation with atom C14 as the flap. The furan ring (O5/C10-C13) makes dihedral angles of 67.43 (12)° and 6.20 (11)° with the mean plane of the dioxolane ring (06/07/C12-C14) and the pyran ring (O1/C1/C6-C9), respectively. The dioxolane ring mean plane makes a dihedral angle of 73.25 (11) ° with the pyran ring. The oxygen atom O2 attached with the pyran ring deviates by -0.0219 (2)Å. The methyl carbon atoms C15 and C16 attached with the mean plane of the dioxolane ring deviate by -0.7157 (3)Å and 1.6682 (3)Å, respectively.

In the crystal, molecules are linked via C-H···O hydrogen bonds into chains along [010], containing R_2_^2^(9) loops (Table 1 and Fig. 2). They stack along the a axis with π-π interactions involving the 4H-chromene units, with centroid-to-centroid distances of 3.6389 (13) Å for Cg1-Cg2^i^ and 3.6555 (13) Å for Cg1···Cg2^ii^, where Cg1 and Cg2 are the centroids of rings O1/C1/C6-C9 and C1-C6, respectively; symmetry codes: (i) x+1/2, -y+1/2, -z+1; (ii) x-1/2, -y+1/2, -z+1.

### Experimental

Triethylamine (1.10 mL, 4 equiv) was added to a stirred solution of 4-hydroxycoumarin (0.32 g, 2 mmol) and(E)-6-(benzyloxy)-2,2-dimethyl -5-(2-nitrovinyl)tetrahydrofuro[3,2-d][1,3]dioxole (0.65 g, 4 mmol) in ally alcohol (6 mL). The reaction mixture was heated at 343 - 353 K for 24h, and the progress of the reaction was monitored by TLC. After completion of the reaction, the solvent was evaporated in vacuum. The resulting residue was further purified by flash column chromatography (ethyl acetate/hexane) on silica gel. Single crystals suitable for X-ray diffraction were obtained by slow evaporation of a solution of the title compound in ethyl acetate at room temperature.

### Refinement

The terminal atoms, C19 and C20, of the allyl acetate group are disordered over two positions (C19/C19' & C20/C20') with a refined occupancy ratio of 0.717 (6):0.283 (6). The C-C distances of the disordered atoms were restrained to be equal. The displacement parameters of the disordered atoms were restrained to be equal for bonded atoms.The hydrogen atoms were placed in calculated positions and treated as riding atoms: C—H = 0.93 - 0.98 Å, with U_iso_(H) = 1.5U_eq_(C) for methyl H atoms and = 1.2U_eq_(C) for other H atoms.

### Crystal data

**Table:**

C_20_H_18_O_7_	*F*(000) = 776
*M**_r_* = 370.34	*D*_x_ = 1.374 Mg m^−^^3^
Orthorhombic, *P*2_1_2_1_2_1_	Mo *K*α radiation, λ = 0.71073 Å
Hall symbol: P 2ac 2ab	Cell parameters from 4459 reflections
*a* = 6.9461 (6) Å	θ = 1.8–28.4°
*b* = 15.5688 (11) Å	µ = 0.11 mm^−^^1^
*c* = 16.5572 (11) Å	*T* = 293 K
*V* = 1790.5 (2) Å^3^	Block, colourless
*Z* = 4	0.30 × 0.25 × 0.20 mm

### Data collection

**Table:**

Bruker SMART APEXII area-detector diffractometer	4459 independent reflections
Radiation source: fine-focus sealed tube	2758 reflections with *I* > 2σ(*I*)
Graphite monochromator	*R*_int_ = 0.072
ω and φ scans	θ_max_ = 28.4°, θ_min_ = 1.8°
Absorption correction: multi-scan (*SADABS*; Bruker, 2008)	*h* = −9→9
*T*_min_ = 0.969, *T*_max_ = 0.979	*k* = −18→20
17828 measured reflections	*l* = −21→22

### Refinement

**Table:**

Refinement on *F*^2^	Primary atom site location: structure-invariant direct methods
Least-squares matrix: full	Secondary atom site location: difference Fourier map
*R*[*F*^2^ > 2σ(*F*^2^)] = 0.045	Hydrogen site location: inferred from neighbouring sites
*wR*(*F*^2^) = 0.127	H-atom parameters constrained
*S* = 1.01	*w* = 1/[σ^2^(*F*_o_^2^) + (0.0549*P*)^2^ + 0.1102*P*] where *P* = (*F*_o_^2^ + 2*F*_c_^2^)/3
4459 reflections	(Δ/σ)_max_ < 0.001
252 parameters	Δρ_max_ = 0.16 e Å^−^^3^
3 restraints	Δρ_min_ = −0.18 e Å^−^^3^

### Special details

**Table:**

Geometry. All esds (except the esd in the dihedral angle between two l.s. planes) are estimated using the full covariance matrix. The cell esds are taken into account individually in the estimation of esds in distances, angles and torsion angles; correlations between esds in cell parameters are only used when they are defined by crystal symmetry. An approximate (isotropic) treatment of cell esds is used for estimating esds involving l.s. planes.
Refinement. Refinement of F^2^ against ALL reflections. The weighted R-factor wR and goodness of fit S are based on F^2^, conventional R-factors R are based on F, with F set to zero for negative F^2^. The threshold expression of F^2^ > 2sigma(F^2^) is used only for calculating R-factors(gt) etc. and is not relevant to the choice of reflections for refinement. R-factors based on F^2^ are statistically about twice as large as those based on F, and R- factors based on ALL data will be even larger.

### Fractional atomic coordinates and isotropic or equivalent isotropic displacement parameters (Å^2^)

**Table:**

	*x*	*y*	*z*	*U*_iso_*/*U*_eq_	Occ. (<1)
C1	0.8806 (3)	0.19806 (13)	0.54128 (13)	0.0478 (5)	
C2	0.8795 (3)	0.13352 (16)	0.48303 (14)	0.0610 (6)	
H2	0.8815	0.0760	0.4981	0.073*	
C3	0.8754 (4)	0.15601 (18)	0.40384 (16)	0.0680 (7)	
H3	0.8729	0.1135	0.3644	0.082*	
C4	0.8749 (4)	0.2410 (2)	0.38153 (14)	0.0708 (7)	
H4	0.8736	0.2554	0.3270	0.085*	
C5	0.8762 (3)	0.30481 (17)	0.43860 (14)	0.0601 (6)	
H5	0.8751	0.3621	0.4226	0.072*	
C6	0.8792 (3)	0.28392 (13)	0.52099 (12)	0.0483 (5)	
C7	0.8791 (3)	0.34926 (13)	0.58404 (14)	0.0490 (5)	
C8	0.8760 (3)	0.31570 (12)	0.66668 (12)	0.0450 (4)	
C9	0.8759 (3)	0.23043 (12)	0.68069 (12)	0.0448 (4)	
C10	0.8641 (3)	0.18772 (13)	0.75846 (12)	0.0474 (5)	
C11	0.8519 (3)	0.10532 (14)	0.77512 (13)	0.0563 (6)	
H11	0.8573	0.0611	0.7374	0.068*	
C12	0.8280 (3)	0.09404 (15)	0.86374 (14)	0.0579 (6)	
H12	0.9320	0.0591	0.8866	0.069*	
C13	0.8357 (3)	0.18598 (15)	0.89523 (13)	0.0559 (5)	
H13	0.9438	0.1934	0.9326	0.067*	
C14	0.5271 (3)	0.13462 (15)	0.91078 (15)	0.0582 (6)	
C15	0.4091 (4)	0.10992 (19)	0.98298 (17)	0.0770 (8)	
H15A	0.3295	0.1575	0.9987	0.116*	
H15B	0.4930	0.0948	1.0268	0.116*	
H15C	0.3292	0.0616	0.9696	0.116*	
C16	0.4054 (4)	0.1653 (2)	0.84136 (17)	0.0866 (9)	
H16A	0.3285	0.2132	0.8585	0.130*	
H16B	0.3228	0.1196	0.8237	0.130*	
H16C	0.4873	0.1828	0.7976	0.130*	
C17	0.8718 (3)	0.38088 (13)	0.73205 (13)	0.0521 (5)	
C18	0.6630 (5)	0.4621 (2)	0.81426 (19)	0.0953 (10)	
H18A	0.6832	0.5197	0.7938	0.114*	0.717 (6)
H18B	0.7520	0.4521	0.8584	0.114*	0.717 (6)
H18C	0.7873	0.4735	0.8388	0.114*	0.283 (6)
H18D	0.6207	0.5150	0.7891	0.114*	0.283 (6)
O1	0.8825 (2)	0.17128 (9)	0.62034 (8)	0.0526 (4)	
O2	0.8790 (2)	0.42692 (10)	0.57063 (10)	0.0661 (4)	
O3	1.0102 (3)	0.41371 (12)	0.76106 (12)	0.0808 (6)	
O4	0.6918 (2)	0.39953 (12)	0.75106 (11)	0.0700 (5)	
O5	0.8584 (2)	0.24039 (9)	0.82508 (9)	0.0600 (4)	
O6	0.6630 (2)	0.19952 (9)	0.93415 (9)	0.0576 (4)	
O7	0.6435 (2)	0.06344 (9)	0.88869 (10)	0.0658 (5)	
C19	0.4667 (7)	0.4514 (4)	0.8412 (4)	0.113 (2)	0.717 (6)
H19	0.3771	0.4407	0.8007	0.135*	0.717 (6)
C20	0.4030 (9)	0.4545 (4)	0.9085 (4)	0.127 (2)	0.717 (6)
H20A	0.4847	0.4649	0.9519	0.152*	0.717 (6)
H20B	0.2720	0.4464	0.9172	0.152*	0.717 (6)
C19'	0.5331 (18)	0.4427 (12)	0.8768 (7)	0.113 (2)	0.283 (6)
H19'	0.5573	0.4675	0.9269	0.135*	0.283 (6)
C20'	0.395 (2)	0.3975 (11)	0.8720 (11)	0.127 (2)	0.283 (6)
H20C	0.3645	0.3711	0.8232	0.152*	0.283 (6)
H20D	0.3173	0.3886	0.9170	0.152*	0.283 (6)

### Atomic displacement parameters (Å^2^)

**Table:**

	*U*^11^	*U*^22^	*U*^33^	*U*^12^	*U*^13^	*U*^23^
C1	0.0385 (10)	0.0503 (12)	0.0547 (11)	−0.0032 (10)	0.0064 (10)	0.0001 (10)
C2	0.0558 (13)	0.0592 (14)	0.0681 (14)	−0.0029 (12)	0.0104 (12)	−0.0096 (11)
C3	0.0587 (14)	0.0806 (18)	0.0649 (15)	−0.0089 (14)	0.0150 (13)	−0.0147 (13)
C4	0.0567 (14)	0.101 (2)	0.0543 (13)	−0.0098 (16)	0.0078 (12)	0.0022 (14)
C5	0.0498 (12)	0.0694 (16)	0.0613 (14)	−0.0040 (12)	0.0034 (11)	0.0117 (12)
C6	0.0363 (10)	0.0534 (12)	0.0552 (12)	−0.0036 (9)	0.0037 (10)	0.0065 (9)
C7	0.0378 (10)	0.0440 (12)	0.0652 (13)	−0.0029 (9)	0.0030 (10)	0.0102 (10)
C8	0.0368 (10)	0.0390 (11)	0.0591 (11)	−0.0031 (9)	0.0010 (9)	0.0031 (9)
C9	0.0391 (10)	0.0402 (11)	0.0551 (11)	−0.0023 (9)	0.0046 (10)	0.0014 (9)
C10	0.0457 (11)	0.0405 (11)	0.0559 (11)	0.0014 (9)	0.0013 (10)	0.0009 (9)
C11	0.0634 (14)	0.0384 (11)	0.0670 (13)	0.0082 (11)	0.0120 (11)	0.0043 (10)
C12	0.0576 (13)	0.0470 (13)	0.0691 (14)	0.0098 (10)	0.0112 (11)	0.0136 (11)
C13	0.0568 (13)	0.0558 (13)	0.0552 (12)	−0.0033 (11)	−0.0054 (10)	0.0083 (11)
C14	0.0545 (12)	0.0504 (13)	0.0697 (14)	0.0003 (11)	0.0034 (12)	−0.0100 (11)
C15	0.0736 (16)	0.0735 (17)	0.0841 (17)	−0.0069 (14)	0.0211 (14)	−0.0063 (15)
C16	0.0611 (16)	0.113 (2)	0.0854 (19)	0.0091 (15)	−0.0148 (14)	−0.0031 (17)
C17	0.0566 (13)	0.0391 (11)	0.0607 (13)	−0.0057 (10)	0.0023 (11)	0.0062 (9)
C18	0.119 (3)	0.081 (2)	0.0861 (19)	0.0074 (19)	0.027 (2)	−0.0218 (17)
O1	0.0619 (9)	0.0387 (7)	0.0572 (8)	0.0002 (7)	0.0054 (7)	−0.0002 (7)
O2	0.0776 (10)	0.0426 (9)	0.0781 (10)	−0.0026 (8)	0.0005 (9)	0.0134 (7)
O3	0.0786 (12)	0.0649 (11)	0.0988 (14)	−0.0292 (10)	−0.0059 (10)	−0.0146 (10)
O4	0.0706 (11)	0.0701 (11)	0.0694 (10)	0.0107 (9)	0.0055 (9)	−0.0126 (9)
O5	0.0813 (10)	0.0417 (8)	0.0568 (8)	−0.0106 (8)	−0.0030 (9)	0.0032 (7)
O6	0.0622 (9)	0.0487 (9)	0.0618 (9)	−0.0015 (7)	0.0016 (8)	−0.0060 (7)
O7	0.0734 (11)	0.0411 (8)	0.0830 (11)	−0.0022 (8)	0.0250 (9)	0.0000 (8)
C19	0.085 (4)	0.147 (5)	0.106 (5)	0.043 (4)	−0.007 (3)	−0.051 (4)
C20	0.102 (3)	0.155 (6)	0.124 (4)	−0.006 (4)	0.028 (3)	−0.045 (4)
C19'	0.085 (4)	0.147 (5)	0.106 (5)	0.043 (4)	−0.007 (3)	−0.051 (4)
C20'	0.102 (3)	0.155 (6)	0.124 (4)	−0.006 (4)	0.028 (3)	−0.045 (4)

### Geometric parameters (Å, º)

**Table:**

C1—O1	1.374 (2)	C14—O7	1.420 (3)
C1—C6	1.378 (3)	C14—O6	1.436 (3)
C1—C2	1.393 (3)	C14—C15	1.500 (3)
C2—C3	1.357 (4)	C14—C16	1.505 (4)
C2—H2	0.9300	C15—H15A	0.9600
C3—C4	1.373 (4)	C15—H15B	0.9600
C3—H3	0.9300	C15—H15C	0.9600
C4—C5	1.371 (4)	C16—H16A	0.9600
C4—H4	0.9300	C16—H16B	0.9600
C5—C6	1.403 (3)	C16—H16C	0.9600
C5—H5	0.9300	C17—O3	1.190 (3)
C6—C7	1.458 (3)	C17—O4	1.322 (3)
C7—O2	1.229 (2)	C18—C19'	1.407 (10)
C7—C8	1.465 (3)	C18—O4	1.443 (3)
C8—C9	1.348 (3)	C18—C19	1.444 (6)
C8—C17	1.484 (3)	C18—H18A	0.9700
C9—O1	1.360 (2)	C18—H18B	0.9700
C9—C10	1.452 (3)	C18—H18C	0.9700
C10—C11	1.315 (3)	C18—H18D	0.9700
C10—O5	1.375 (2)	C19—C20	1.200 (7)
C11—C12	1.487 (3)	C19—H19	0.9300
C11—H11	0.9300	C20—H20A	0.9300
C12—O7	1.429 (3)	C20—H20B	0.9300
C12—C13	1.524 (3)	C19'—C20'	1.194 (10)
C12—H12	0.9800	C19'—H19'	0.9300
C13—O6	1.378 (3)	C20'—H20C	0.9300
C13—O5	1.446 (3)	C20'—H20D	0.9300
C13—H13	0.9800		
			
O1—C1—C6	121.77 (19)	C14—C15—H15A	109.5
O1—C1—C2	116.2 (2)	C14—C15—H15B	109.5
C6—C1—C2	122.1 (2)	H15A—C15—H15B	109.5
C3—C2—C1	118.9 (2)	C14—C15—H15C	109.5
C3—C2—H2	120.6	H15A—C15—H15C	109.5
C1—C2—H2	120.6	H15B—C15—H15C	109.5
C2—C3—C4	120.5 (2)	C14—C16—H16A	109.5
C2—C3—H3	119.7	C14—C16—H16B	109.5
C4—C3—H3	119.7	H16A—C16—H16B	109.5
C5—C4—C3	120.8 (2)	C14—C16—H16C	109.5
C5—C4—H4	119.6	H16A—C16—H16C	109.5
C3—C4—H4	119.6	H16B—C16—H16C	109.5
C4—C5—C6	120.2 (2)	O3—C17—O4	125.0 (2)
C4—C5—H5	119.9	O3—C17—C8	124.9 (2)
C6—C5—H5	119.9	O4—C17—C8	110.01 (18)
C1—C6—C5	117.5 (2)	C19'—C18—O4	118.5 (8)
C1—C6—C7	120.15 (18)	O4—C18—C19	106.1 (3)
C5—C6—C7	122.3 (2)	C19'—C18—H18A	123.3
O2—C7—C6	123.8 (2)	O4—C18—H18A	110.5
O2—C7—C8	121.3 (2)	C19—C18—H18A	110.5
C6—C7—C8	114.84 (17)	C19'—C18—H18B	79.6
C9—C8—C7	120.80 (19)	O4—C18—H18B	110.5
C9—C8—C17	123.24 (18)	C19—C18—H18B	110.5
C7—C8—C17	115.96 (17)	H18A—C18—H18B	108.7
C8—C9—O1	122.74 (18)	C19'—C18—H18C	107.6
C8—C9—C10	127.15 (18)	O4—C18—H18C	107.7
O1—C9—C10	110.10 (16)	C19—C18—H18C	137.1
C11—C10—O5	114.30 (18)	H18A—C18—H18C	81.3
C11—C10—C9	129.54 (19)	C19'—C18—H18D	107.7
O5—C10—C9	116.12 (16)	O4—C18—H18D	107.7
C10—C11—C12	109.2 (2)	C19—C18—H18D	86.8
C10—C11—H11	125.4	H18B—C18—H18D	130.8
C12—C11—H11	125.4	H18C—C18—H18D	107.1
O7—C12—C11	115.1 (2)	C9—O1—C1	119.63 (16)
O7—C12—C13	104.20 (17)	C17—O4—C18	116.9 (2)
C11—C12—C13	102.87 (17)	C10—O5—C13	107.35 (15)
O7—C12—H12	111.4	C13—O6—C14	109.78 (16)
C11—C12—H12	111.4	C14—O7—C12	108.99 (16)
C13—C12—H12	111.4	C20—C19—C18	129.0 (6)
O6—C13—O5	112.41 (18)	C20—C19—H19	115.5
O6—C13—C12	105.87 (18)	C18—C19—H19	115.5
O5—C13—C12	106.21 (17)	C19—C20—H20A	120.0
O6—C13—H13	110.7	C19—C20—H20B	120.0
O5—C13—H13	110.7	H20A—C20—H20B	120.0
C12—C13—H13	110.7	C20'—C19'—C18	126.4 (14)
O7—C14—O6	104.14 (16)	C20'—C19'—H19'	116.8
O7—C14—C15	108.5 (2)	C18—C19'—H19'	116.8
O6—C14—C15	108.96 (19)	C19'—C20'—H20C	120.0
O7—C14—C16	111.8 (2)	C19'—C20'—H20D	120.0
O6—C14—C16	110.6 (2)	H20C—C20'—H20D	120.0
C15—C14—C16	112.5 (2)		
			
O1—C1—C2—C3	179.2 (2)	O7—C12—C13—O5	−119.41 (19)
C6—C1—C2—C3	−0.5 (3)	C11—C12—C13—O5	1.1 (2)
C1—C2—C3—C4	0.8 (4)	C9—C8—C17—O3	93.4 (3)
C2—C3—C4—C5	−0.8 (4)	C7—C8—C17—O3	−87.0 (3)
C3—C4—C5—C6	0.3 (4)	C9—C8—C17—O4	−89.1 (2)
O1—C1—C6—C5	−179.62 (18)	C7—C8—C17—O4	90.6 (2)
C2—C1—C6—C5	0.1 (3)	C8—C9—O1—C1	−3.0 (3)
O1—C1—C6—C7	−0.2 (3)	C10—C9—O1—C1	175.57 (17)
C2—C1—C6—C7	179.58 (19)	C6—C1—O1—C9	2.4 (3)
C4—C5—C6—C1	0.0 (3)	C2—C1—O1—C9	−177.38 (16)
C4—C5—C6—C7	−179.5 (2)	O3—C17—O4—C18	−2.7 (3)
C1—C6—C7—O2	179.6 (2)	C8—C17—O4—C18	179.8 (2)
C5—C6—C7—O2	−1.0 (3)	C19'—C18—O4—C17	−131.5 (8)
C1—C6—C7—C8	−1.4 (3)	C19—C18—O4—C17	−162.3 (3)
C5—C6—C7—C8	178.04 (18)	C11—C10—O5—C13	−1.2 (3)
O2—C7—C8—C9	179.9 (2)	C9—C10—O5—C13	176.71 (18)
C6—C7—C8—C9	0.8 (3)	O6—C13—O5—C10	−115.4 (2)
O2—C7—C8—C17	0.3 (3)	C12—C13—O5—C10	0.0 (2)
C6—C7—C8—C17	−178.81 (17)	O5—C13—O6—C14	99.2 (2)
C7—C8—C9—O1	1.4 (3)	C12—C13—O6—C14	−16.4 (2)
C17—C8—C9—O1	−179.03 (18)	O7—C14—O6—C13	26.2 (2)
C7—C8—C9—C10	−176.96 (18)	C15—C14—O6—C13	141.8 (2)
C17—C8—C9—C10	2.7 (3)	C16—C14—O6—C13	−94.0 (2)
C8—C9—C10—C11	175.5 (2)	O6—C14—O7—C12	−25.7 (2)
O1—C9—C10—C11	−3.0 (3)	C15—C14—O7—C12	−141.66 (19)
C8—C9—C10—O5	−2.1 (3)	C16—C14—O7—C12	93.7 (2)
O1—C9—C10—O5	179.38 (16)	C11—C12—O7—C14	−95.8 (2)
O5—C10—C11—C12	2.0 (3)	C13—C12—O7—C14	16.0 (2)
C9—C10—C11—C12	−175.6 (2)	C19'—C18—C19—C20	21.5 (15)
C10—C11—C12—O7	110.8 (2)	O4—C18—C19—C20	140.6 (7)
C10—C11—C12—C13	−1.8 (3)	O4—C18—C19'—C20'	−29 (2)
O7—C12—C13—O6	0.3 (2)	C19—C18—C19'—C20'	44.0 (15)
C11—C12—C13—O6	120.75 (19)		

### Hydrogen-bond geometry (Å, º)

**Table:**

*D*—H···*A*	*D*—H	H···*A*	*D*···*A*	*D*—H···*A*
C11—H11···O3^i^	0.93	2.47	3.190 (3)	134
C12—H12···O2^i^	0.98	2.54	3.477 (3)	160

Symmetry code: (i) −*x*+2, *y*−1/2, −*z*+3/2.
